# Functional Drug Screening in the Era of Precision Medicine

**DOI:** 10.3389/fmed.2022.912641

**Published:** 2022-07-08

**Authors:** Giulia C. Napoli, William D. Figg, Cindy H. Chau

**Affiliations:** Molecular Pharmacology Section, Genitourinary Malignancies Branch, Center for Cancer Research, National Cancer Institute, National Institutes of Health, Bethesda, MD, United States

**Keywords:** functional medicine, drug screening, chemosensitivity, precision oncology, organoid

## Abstract

The focus of precision medicine is providing the right treatment to each unique patient. This scientific movement has incited monumental advances in oncology including the approval of effective, targeted agnostic therapies. Yet, precision oncology has focused largely on genomics in the treatment decision making process, and several recent clinical trials demonstrate that genomics is not the only variable to be considered. Drug screening in three dimensional (3D) models, including patient derived organoids, organs on a chip, xenografts, and 3D-bioprinted models provide a functional medicine perspective and necessary complement to genomic testing. In this review, we discuss the practicality of various 3D drug screening models and each model’s ability to capture the patient’s tumor microenvironment. We highlight the potential for enhancing precision medicine that personalized functional drug testing holds in combination with genomic testing and emerging mathematical models.

## Introduction

Precision medicine has become synonymous with genomic medicine (1, 2), yet unfortunately genomics-guided medicine is not available to all patients, nor is therapeutic success guaranteed. Recent progress in precision medicine guided by genetic biomarkers has inspired the development of tumor mutation databases such as OncoKB, which is the first somatic human cancer variant database recognized as a source of precision oncology knowledge by the United States Food and Drug Administration (FDA) in 2021 (3). Likewise, several genotype-guided therapies have been approved and new pan-cancer sequencing panels (e.g., MSK-impact) have been developed to expand the applicability of genome driven cancer treatment. However, the ground-breaking precision medicine NCI-MATCH trial, one of the first major trials to assign treatment based on genetic features instead of cancer type, was able to assign only 17.8% of the cohort to a treatment arm (4). In the SHIVA trial, 41% of patients lacked a targetable molecular alteration and no improvement was observed in the progression free survival (PFS) of patients with metastatic solid tumors treated with molecularly targeted agents compared to standard of care treatment (5). Similarly, the SIGNATURE program of eight phase 2 agent-specific basket protocols observed only a 17% clinical benefit rate (i.e., partial response, complete response, or stable disease) among those treated with biomarker guided therapies (6). Ultimately, an estimated 7% of cancer patients benefit from genome driven therapies (7), excluding a vast majority of patients from most of the progress achieved thus far by precision medicine.

As illustrated by the SHIVA trial and SIGNATURE program discussed above, possession of an actionable mutation and eligibility for genome-matched treatment aside, biomarker presence does not alone guarantee therapeutic success. Many clinically important driver mutations are therapeutic targets (BRAF, PIK3CA/MTOR, ALK, and EGFR) present in both benign and malignant conditions (8), and a recent study observed limited evolution of the driver mutation profile in metastatic cancer patients under therapeutic pressure, highlighting the urgency that functional medicine look beyond genomics to uncover the underpinnings of disease progression (9). Functional precision oncology proposes a comprehensive approach, coupling genomics with clinical knowledge and functional assays (1), and focusing on the phenotypic behavior of the patient’s disease in a faithfully modeled treatment setting. Ultimately, precision medicine’s progress thus far has taught us that genotype alone does not reliably inform drug response, and that the incorporation of functional assays, particularly high-throughput drug screening, is necessary for the progression of precision medicine.

In this review, we provide an overview of functional precision medicine, discuss the technological advances of tumor models for drug testing, and highlight the latest clinical trials that incorporate genomics and functional drug screening. We searched ClinicalTrials.gov for relevant trials using the terms: next-generation sequencing (NGS), or drug screening AND organoid, PDO, xenograft, PDX, patient-derived, organ on a chip, OOC, 3D bioprinted, microfluidic, drug screen. We also included trials cited in relevant articles that we judged to be important. We searched PubMed for reviews and original research papers published between 1 January 2015 and 1 February 2022 using the keywords: chemosensitivity, cancer organoids, cancer xenografts, organ on a chip, microfluidic cancer model, 3D printed, 3D bioprinted, patient-derived organoids, patient-derived cancer xenografts, patient-derived organ on a chip, functional precision medicine, functional precision oncology, metastasis models, and cancer drug screening models. We incorporated publications cited in the articles from our search that we judged to be important. The reference list was generated on the basis of ingenuity and pertinence to the topic of this review.

## History of Drug Screening *via* Chemosensitivity Assays

The first report of drug sensitivity testing using primary tumor tissues from patients to predict response to chemotherapeutic agents was published over 60 years ago by Dr. Jane C. Wright, an African-American physician and the only female founding member of the American Society of Clinical Oncology (ASCO) (10). Wright’s model for testing and selecting cancer therapeutics based on the responsiveness of individual tumors, subsequently referred to as chemotherapy sensitivity and resistance assays (CSRAs), was conceptually similar to antibiotic sensitivity testing and represented one of the earliest steps toward personalized medicine in oncology.

Over the next few decades, various *in vitro* renditions of CSRAs, including the human tumor stem cell assay (11), were developed but met limited success due to variability in responses and lack of reproducibility (12). Chemosensitivity tests involve dissociation of patient tumor tissue, followed by primary culture of these tumor cells, and finally assessment of cell response to various treatments. Several commercial tests are available, including ChemoID and the Oncogramme. Chemosensitivity testing involves culturing a patient’s cells in monolayers, which fails to truly recapitulate the microenvironment in which the tumor grows and evolves, including the three-dimensional (3D) setting, immune system, signaling intermediates, heterogeneity, and architecture of the tumor (13, 14). Likewise, cell morphology, proliferation, differentiation, and drug metabolism are significantly different in 2D versus 3D culture (15). Twenty-seven strains of the MCF7 breast cancer cell line grown in two laboratories displayed rapid genetic diversification over the culture process as well as considerably different drug responses, exposing substantial genetic and molecular variation in cell lines (16). The efficacy and potency of 10 lung cancer treatments demonstrated significant differences in 3D versus 2D environments (17). Furthermore, human epidermal growth factor receptor has been observed to form heterodimers in 2D and homodimers in spheroids, altering HER2 activation, PI3K signaling, and response to trastuzumab, thus effecting the authenticity of 2D models (18). Drug screening should thus be performed in a three-dimensional setting to replicate the disease environment and cellular response to therapeutics. To date, the use of CSRAs is not recommended in routine clinical practice but rather in a clinical trial setting (19) until technological advances can develop reliable and robust drug screening assays capable of accurately predicting tumor response to chemotherapy and targeted agents.

## Role of the Tumor Model

**FIGURE 1:**
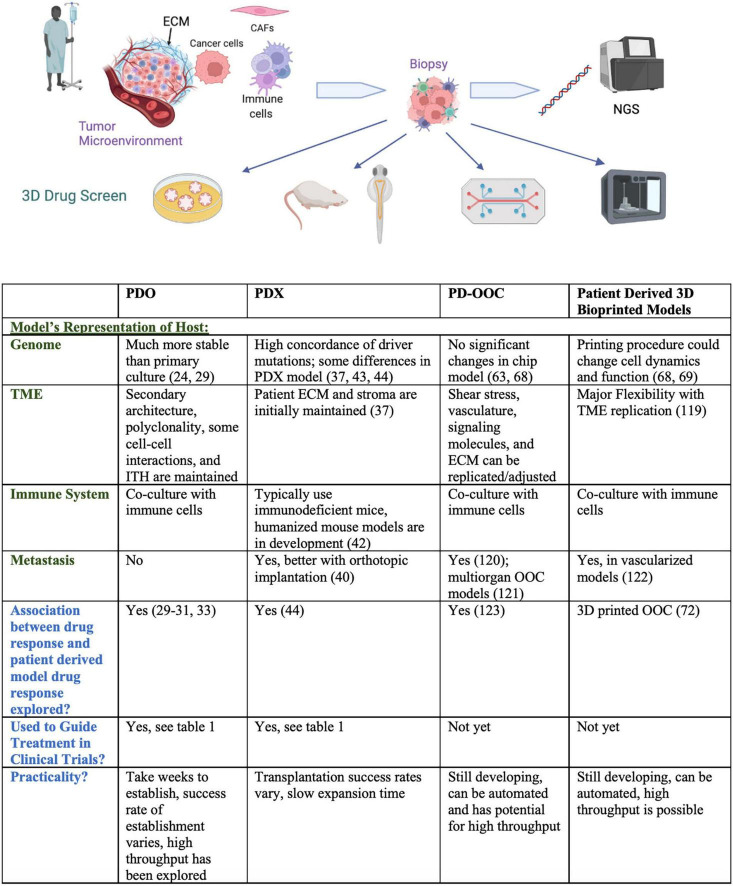
Comparison of 3D patient-derived drug screening platforms. Pictogram was created with Biorender.com.

The tumor model ideally mimics the tumor microenvironment in a 3D, tissue specific setting and encompasses the genomic characteristics of the tumor as well as the host’s immune system. The tumor stroma consists of the extracellular matrix (ECM), immune cells, cancer associated fibroblasts, and angiogenic vascular cells among others. The stromal cells interact with each other and tumor cells by secreting chemokines, growth factors, and regulatory miRNAs, all implicit in cancer progression (20). Endothelial cells are responsible for neovascularization, which enables new tumor formation, while infiltrating immune cells supply growth mediators, encourage neoplastic cell proliferation, and modify the ECM (21). ECM proteins impact cell behavior, and changes in ECM components contribute to hypoxia and several other hallmarks of cancer. Integrins, the main cellular adhesion receptors, often display altered expression in tumors and play a wide variety of roles in cancer progression and the definition of TME characteristics (22). Finally, cancer associated fibroblasts (CAFs) secrete signaling proteins that stimulate cancer cell proliferation, participate in the epithelial to mesenchymal transition (implicated in metastasis), and express proinflammatory mediators that recruit immune cells. Ultimately, components of the tumor stroma aid cancer cells in the evasion of cell death as well as induction of angiogenesis and metastasis (21), and are thus necessary components of preclinical drug testing and disease models. Furthermore, as single-cell RNA sequencing has revealed the extent of intratumoral heterogeneity and allowed for the identification of inflammatory, proangiogenic, treatment resistant, and pro-metastatic subpopulations (23), tumor models must utilize patient-derived specimens that encompass each individual’s disease profile and heterogeneity. In the next sections, we describe 3D tumor models developed for drug screening. A comparison of 3D patient-derived drug screening platforms is provided in Figure 1.

## Patient-Derived Organoids

patient-derived organoids (PDOs) are derived from patient tissue embedded in a matrix, cultured in suspension, or grown as a co-culture model. Organoids are a 3D alternative to 2D cell culture that better represent the tumor microenvironment (cell morphology and viability, drug metabolism and secretion) (24). PDOs bridge 2D models with patient-derived xenografts (PDX) and are established by mincing patient tissue and plating cells with nutrient rich media on a basement membrane-mimicking substance (e.g., matrigel). PDOs can be treated, cryopreserved, and further expanded for downstream analyses within weeks of initial culture all while remaining genetically stable (25). PDOs also retain various features of the original tumor, including secondary architecture, polyclonality, and intratumoral heterogeneity. Optical metabolic imaging (OMI) can be employed to examine metabolic activity at the single cell level in PDOs, which could help identify treatment responsive and non-responsive subpopulations (26). CRISPR-Cas9 genome editing has also been used in the organoid model to identify tumor suppressors as well as drivers of TGF-β resistance (27, 28). Gene expression profiles have been shown to remain more stable in PDOs compared to 2D cell culture (24) and one study in gastrointestinal cancer observed 88% accuracy of PDOs in predicting patient treatment response (29). Rectal cancer PDOs have displayed a range of responses to chemoradiation, and oncogenic tumor mutations and response to chemoradiation have shown great similarity in rectal cancer patient/organoid pairs (30, 31). Genomic, transcriptomic, and therapeutic profiling (also referred to as pharmacotyping) of pancreatic PDOs uncovered novel driver genes, transcriptional signatures that might predict drug response, and alternative targeted therapies for chemo-resistant PDOs (32). A 2021 review pooled the sensitivity and specificity values of PDO screening for predicting patient treatment response from 17 studies and revealed the promising accuracy of this model (33). Though PDOs may lack an immune environment, they can be co-cultured with immune cells to mimic the human immune system (34). Other downsides of the PDO model are the 4–6 weeks required to establish organoids (35), and establishment success rates ranging from 16 to 100% (33). In addition to the low organoid formation rate, the absence of stromal cells in many organoid models presents a major limitation.

A model that falls beside patient derived organoids is the patient-derived explant (PDE) model. PDEs are generated when freshly resected tissue is promptly submerged in media or grown on a matrix or sponge in media and used for drug screening, thus preserving the original tumor architecture and intact stroma. The Histoculture Drug Response Assay (HDRA) and other explant platforms were successful in 1990s and 2000s, but have not advanced since then, likely because patient-derived explants only survive intact for short periods of time (36).

## Patient-Derived Xenografts

Patient-derived xenografts (PDXs) typically involve implanting patient tumor cells in immunodeficient mice; immunocompromised mice are necessary to prevent rejection of human tumor cells by the host animal’s immune system and improve engraftment success rates (37). Tumor cells are implanted subcutaneously or orthotopically, and tumor growth as well as drug response is followed using calipers and fluorescent or bioluminescent imaging, along with pharmacokinetic and pharmacodynamic parameters (37). Implantation technique is important to consider as morphologic, interactome, and metabolic differences have been observed between tumors implanted orthotopically and those implanted subcutaneously (38, 39). One major benefit of the PDX model is that the patient’s stroma is initially preserved and implanted into the mouse model, though it is ultimately replaced with a murine stroma over time (37). The PDX model provides a 3D *in vivo* perspective, recapitulates tumor heterogeneity, and allows for measurement of pharmacokinetic and pharmacodynamic parameters.

Shortcomings of this approach are expense, low transplantation success rate, the large number of tumor samples needed, and the time required for xenografts to grow. Transplantation success rates vary, ranging from 20 to 100% in osteosarcoma (40) and 11 to 100% in head and neck cancer, and depend on whether specimens were obtained from a primary or metastatic site (41). Likewise, in some cancer types, metastasis is not observed *in vivo* and some features of the tumor are not fully represented including the TME, tumor-host interactions, and local growth of PDX’s (40). The use of immunodeficient mice fails to mimic the role of the immune system in patient tumors and specifically poses difficulty when testing immunotherapies against human-specific immune checkpoints (42). Some humanized mouse models have been developed, maintaining lineages of human blood cells in the animal model and mimicking the immune environment (34). Despite the relative closeness to the human TME that PDX models offer, mouse specific tumor evolution has been observed in PDX models. Likewise, PDXs may not better represent the genomics of primary tumors compared to cell lines, and propagation in PDXs has been shown to distance the CNA landscapes of PDXs from those of the primary tumors from which they were derived (43). A study comparing the genomic landscapes of 536 PDX models with their parent tumors revealed high tumor purity and sub-tumor clone selection in PDXs compared to primary human tumors, high concordance of driver mutations in PDXs and their matched counterparts, and downregulation of mutated tumor suppressor genes in PDX models (44). Although PDX models do not fully recapitulate the mutation profile of the host, advancements in CRISPR are allowing for the induction of driver mutations into the host, establishing somatically engineered mouse models. The introduction of genomic modifications by homologous recombination in embryonic stem cells is also helping pave the way toward genetically engineered mouse models (37). Many groups have begun PDX compilation projects, including EurOPDX consortium and the ITCC-P4 project. The NCI PDXNet Consortium analyzed the inter-laboratory reproducibility of PDX drug studies for three PDX models across four PDX development and trial centers using independently selected standard operating protocols (45). This work revealed that in the context of cytotoxic agents, the PDX model yielded accurate and consistent responses to treatment; the authors also developed standardized PDX biostatistical analysis workflows (45).

PDX-derived organoids (PDXOs) have also been explored as high throughput alternatives to PDXs that allow for prolonged culture and preservation of the unique disease profiles initially cultured *in vivo*. Breast cancer PDXOs have shown similar drug responses and driver mutation profiles to their paired PDX models and demonstrated little change in gene expression over time (46). Pancreatic ductal adenocarcinoma PDXOs displayed similar drug sensitivity and glycan profiles to their PDX counterparts (47). A protocol for deriving a PDXO from a PDX was recently published by Xu and colleagues, and the established colorectal cancer PDXO displayed similar drug sensitivity and gene expression profiles to its parent PDX (48). Although this higher throughput method captures the drug response profiles of PDX counterparts, the time required establish both a PDX and PDO may pose a limit for implementation in the precision oncology realm.

## Zebrafish Patient-Derived Xenografts

The zebrafish patient-derived xenograft model (zPDX) is a high throughput and low-cost alternative to murine models, zebrafish can hatch 150–200 eggs per week, and display a high level of genetic and molecular pathway conservation with humans. Shortcomings of the zebrafish model include high mortality after injection and the difference in body temperature between zebrafish and humans. Xenotransplantation procedures using larval zebrafish recipients are conducted below 37 degrees, and at those temperatures the tumor cells fail to proliferate and form tumors at the same rate that they do in immunocompromised mice or human patients (49, 50). The size of zebrafish also presents a limitation; the number of cells that can be transplanted is restricted to 100–200 and this sample size does not always capture the genetic heterogeneity and thus drug response in human tumors (50). The transparent zebrafish body allows for tracking of fluorescently labeled cells and extracellular vesicles released from tumors (51). Likewise, the behavior of xenografted single cancer cells can be tracked in zebrafish (52, 53) and confocal microscopy can be employed to study several aspects of cancer progression, including cell state transitions and regeneration (54). Compared to mice, zebrafish provide an improved model of pharmacokinetics *in vivo* and drugs can be added directly to the zebrafish habitat (49). Immune deficient zebrafish exist, but if the xenograft is implanted early enough, immunosuppression is not necessary, which serves as an advantage compared to murine PDX models (49). Furthermore, several zebrafish knockout lines and CRISPR systems have been optimized to expand the utility of this model (50).

## Organ-On-a-Chip Applications

Organ-on-a-chip (OOC) technology, also known as microphysiological systems, is a microfluidics-based approach to disease modeling composed of a network of channels that allow continuous perfusion of cells grown on a basement membrane mimicking substance. The microfluidics model allows for simultaneous culture of multiple cell types on the same chip and is designed for precise control of chemical gradients as well as drug, signaling molecule, and media delivery (55). OOC technology has been expanded to multi-organ and human-on-a-chip technology which permit more comprehensive pharmacokinetic and pharmacodynamic studies (56). Recently, a 200 well high throughput microfluidic platform was designed that allows continuous drug delivery programs, vascularization of micro organ and micro tumor models, and a level of standardization that avoids the human error inherent in PDO models. OOCs can be analyzed in real time by the use of fluorescent cellular marker proteins and can be easily harvested from the chamber device for downstream analysis (57). OOC platforms provide the ability to mimic the physical and biochemical profiles of the tumor microenvironment by allowing for calibration of mechanical parameters which are not as tunable in an organoid model. For instance, the shear stress of each respective organ and the stiffness of the ECM, which has been studied as a contributor to pathogenesis in the microfluidics model, can be easily adjusted (58).

In pancreatic cancer, microfluidic chips have been used to study metastasis (59) and humanized microfluidic devices have allowed for drug combination screening (60). Patient-specific glioblastoma (GBM) on a chip models have been developed to explore GBM subtypes and perform personalized screening of immunotherapies and combination therapies for GBM patients (61). Although patient-derived OOC technology has not yet been optimized, this model demonstrates major potential for patient-specific drug screening in a finely tunable and highly representative model of cancer. Likewise, this platform models an immune system, which remains a challenge for PDX models. Several companies have developed commercially available organs on chip, which are typically available as liver or cancer on a chip for use in pharmacokinetic and pharmacodynamic studies (56), however this model must be further refined before advancement to clinical trials.

More recently, OOC technology has been coupled with 2D cell line culture, organoid, and organotypic slice culture methods (62). An OOC platform that enables prolonged growth of organotypic tumor slices, mimicry of blood vessels, and modeling of gradients was examined in breast and prostate cancer models. Gene expression analysis revealed that immune responses were affected under *ex vivo* conditions (which the authors believe to be an effect of increased cellular stress in the *ex vivo* model), but not in the OOC platform. OOC organotypic slice culture response to antiandrogen therapy in prostate cancer and cisplatin treatment in breast cancer modeled drug response in paired PDXs (63). Organotypic slice culture distinguishes itself from other models because the original tissue architecture and tumor heterogeneity are maintained. This combination of PDE-inspired slice culture with OOC technology is promising both in terms of its ability to capture the TME and its potential for reproducibility and adaptation to high throughput screening.

## Bioprinting

3D bioprinting provides another platform for patient-specific disease modeling and drug testing. Briefly, 3D printing involves conceptualization of the model, deconstruction of the product into slices for construction of a code to be read by a 3D printer, formulation of products to be used in the printing process and preparation of the 3D printer. Several printing methods and materials are available and thoroughly reviewed elsewhere (64, 65). Bioprinting utilizes cells to form spheroids or organoids, mimicking vascularization and cellular interactions to faithfully recapitulate disease progression and drug response (66). Cancer bioinks contain patient-derived cancer cells, circulating tumor cells, cancer stem cells, cancer associated fibroblasts, growth medium, and growth factors (64). 3D bioprinting is performed in a scaffold-based or scaffold free approach; in a scaffold-based approach, the 3D form is printed with a bioink consisting of cells in a hydrogel. Meanwhile, in the scaffold free approach, cells are bioprinted on a material mold, like agarose, which is removed once the printed tissue has developed. 3D models have been printed such that matrix stiffness, distribution of biochemical factors, cell migration, biomolecular gradients, and perfusable vascular networks can be imitated and adjusted (67). 3D bioprinting allows for usage of a variety of cell types and precise mimicry of the TME based on cancer type. Unfortunately, bioprinting has been shown to alter the morphology, protein expression, phosphorylation of oncogenes, and gene expression in MCF7 breast cancer cells (68). In a comparison of HepG2 liver cells grown in 2D and 3D printed environments, the expression of cytochromes key to liver function and mRNA were strikingly different. Analysis of mRNA sequencing data suggested that the cell populations had different microenvironments and gene expression was higher in the 3D model compared to the 2D model, indicating that the cells reached higher maturity in 3D conditions. The IC50s of antitumor drugs were significantly higher in 3D models compared to 2D models, and drug response in 3D bioprinted models was more closely correlated to drug response in human clinical trials (69). While 3D bioprinting allows for mimicry of tumor microenvironment diversity and structure, the printing process must be optimized such that cell viability and multi-omics profiles are preserved during the printing process.

The flexibility provided by 3D printing has inspired a range of preclinical experiments. 3D bioprinted models have been utilized to investigate the breast cancer bone metastasis microenvironment (70). 3D printed mini brains have allowed study of the cross talk between tumor cells and macrophages, examination of the epithelial to mesenchymal transition in glioblastoma (GBM), and drug screening (71). A patient-derived GBM on a chip model has been 3D printed to capture the brain ECM microenvironment, the stroma, and an oxygen gradient for patient-specific drug testing. These GBM models were established within a clinically reasonable time frame of 1–2 weeks (72). Beyond 3D printing, 4D bioprinting describes 3D printing performed with smart materials that change shape upon the introduction of heat, light, or a change in pH. A 4D printed cell culture array was designed for drug testing in single cell derived GBM-PDOs which, upon heating, transforms from a cell culture array to a programmable cassette for subsequent histological and immunohistochemical analysis while maintaining the tissue array orientation. PDOs grown in this array recapitulated tumor phenotypes as well as gene expression in GBM patients and the platform used an air liquid interface, modeling elements of the TME and immune environment (73).

## Cell Reprogramming (Conditional Reprogramming and Induced Pluripotent Stem Cells)

Conditional reprogramming (CR) of tumor cells is emerging as another technology with potential in functional precision medicine (FPM). Explained in depth by Liu et al. (74), CR involves reprogramming epithelial cells such that they assume the characteristics of adult stem cells, allowing for timely culture of epithelial cells without genetic manipulation. The technology has been optimized so that growth conditions exclusively support the proliferation of malignant cells, known as individualized-CR (i-CR), because rapid expansion of normal cells complicates interpretation of drug response data (75). In a colorectal cancer cohort, the morphology of cultured i-CR cells remained similar to that of original patient cells, the i-CR cells expanded quickly, allowing for timely pre-clinical testing, and i-CR cells were found to maintain the genetic heterogeneity of the tissues from which they were originally derived. Forty-two percent of patient tumor samples were successfully cultured and for 17 eligible patients, the accordance rate between i-CR tests and clinical response was 94%. Although CR technology alone does not recapitulate the TME and thus cannot be used to screen treatments involving the TME such as immunotherapy or anti-angiogenic agents, it provides a more efficient alternative to traditional 2D cell culture (76). CR cells can also be utilized to establish organoids and xenografts. Wang and colleagues utilized samples from colorectal cancer (CRC) patients to develop PDX models, perform i-CR drug screening on PDX-derived tumor cells, and identify synergistic drug combinations which were subsequently validated in paired PDX models (77). Unfortunately, the CR process results in changes in protein expression and activation or blockage of various signaling pathways, thus limiting the fidelity of this model (78).

Induced pluripotent stem cells (iPSC) have also been considered as a potential study model for preclinical oncology drug testing. iPSCs are somatic cells reprogrammed by four transcription factors such that they can differentiate into multiple cell lineages and organoid culturing of iPSCs mimics tumorigenesis and have been established for multiple organs. iPSCs provide a more reliable source of cells compared to traditional 2D culture and could overcome issues with genetic drift that occur in long term primary culture (79). Despite the potential utility of the iPSC model, this reprogramming, like CR, can result in loss of the drug resistance, metastatic potential, and tumorigenic behaviors of the original cells; likewise, the epigenetic reprogramming implicated in this process may alter the expression of oncogenes and affect several cancer-related pathways (79).

## Beyond the Benchtop: Mathematical Models and Artificial Intelligence

Mathematical models find associations in data sets and allow for data modeling using a variety of biomarkers and patient characteristics. These models can be used to select chemotherapy agents based on computational models of apoptotic pathways and to identify personalized combination treatments based on tumor biopsy data (80). Models must first be trained on existing data and validated with untrained data before they can predict outcomes for existing data sets (81). Recently, a precision oncology framework titled SELECT (synthetic lethality and rescue mediated precision oncology *via* the transcriptome) was developed, which employs the genetic interactions of drug targets to identify individualized therapies for cancer patients. Based on the pre-treatment transcriptomic data of 35 clinical trials using targeted and immunotherapy to treat 10 different cancer types, this framework predicted patient response in 80% of the clinical trials examined, suggesting that transcriptomics could be used to guide precision medicine treatment decision-making (82). In a similar vein, Beyondcell, a computational methodology, utilizes single-cell RNA-seq data to identify “therapeutic clusters” of tumor cells that exhibit similar drug responses. This computational method has correctly predicted responders and non-responders to immunotherapy among melanoma patients (83) and the level of specificity provided could greatly compliment genomic and functional data to guide treatment decision making.

Machine learning (ML) models are similar to mathematical models and are based on the concept of artificial intelligence (AI) that computers learn through experience and can track and predict drug response, cancer risk, and cancer outcomes over time. ML is unique because multiple datasets can be integrated, including genomic, transcriptomic, proteomic, and image-based information to identify trends across the datasets. ML algorithms can recognize patterns not necessarily evident to the human eye and identify new biomarkers (84). ML is broken down into unsupervised and supervised learning in which clusters and patterns are identified automatically or based on labeled data. Once patterns are recognized, they can be used to understand patient datasets and predict outcomes. Artificial neural networks, or deep neural networks, are composed of an input, an output, and many connections in between that mimic the brain’s connectivity and can process complex images or identify spatial and temporal features in a dataset (85). Despite the immense power of mathematical and machine learning models, data management, storage, and accessibility are important to consider. Missing data, the cost of data acquisition, the need for data preprocessing, and integration of large amounts of diverse data pose challenges to the developing field. Furthermore, interpretation of the conclusions garnered from mathematical and machine learning approaches remains a challenge and patterns revealed by AI cannot always be elucidated by our current scientific knowledge base. While ML has the power to unify and synthesize data points on each patient and their individual tumor profile, ML must be implemented carefully as a complement to physician knowledge (86).

Machine learning has been applied to accurately predict the efficacy of molecularly targeted and non-specific chemotherapy drugs in cancer cell lines and shows promise for translation into the clinical realm (87). A deep convolutional neural network has been developed which predicts immunotherapy response using ML classification of histology slide images with clinico-demographic data and could provide clinicians with patient risk levels (88). Several companies have produced FDA-approved artificial intelligence software offering image-based AI-guided evaluation of pathology slides and MRI/CT scans. Recently, a plasma lipidomics and ML platform named the Lung Cancer Artificial Intelligence Detector (LCAID) was developed for early stage lung cancer detection and identified early stage lung cancer with high specificity and sensitivity, providing a time efficient and minimally invasive diagnostic option (89). Though mathematical and ML models have primarily been explored in the diagnostic and predictive realms, they have major potential in the clinical arena as well. Recent machine learning methods have incorporated AI computational modeling with gene expression panels such as the OncotypeDx. OncotypeDx is a genomics-based 21-gene Recurrence Score assay that has been validated for prognosis and prediction of chemotherapy benefit or recurrence in early stage, ER+ breast cancer (90). The assay has been used to guide treatment decisions, is widely included in clinical trials, and its implementation is recommended in ASCO and NCCN treatment guidelines (91, 92). Omics-based assays and big data analysis have paved the way for personalized medicine as a whole and in knowledge-based mathematical/AI models.

## Precision Oncology Trials Incorporating Functional Precision Medicine

Despite the impressive development of the aforementioned models, these technologies have yet to enter oncology clinical trials with great force for personalized drug screening. Many feasibility trials are ongoing for PDO (NCT03577808, NCT03544255, NCT04261192, NCT03979170, NCT03453307, NCT03655015, NCT03990675, NCT04371198, and NCT03890614) and PDX (NCT02124902, NCT02283658, NCT02597738, NCT04724070, NCT02752932, and NCT02616211) models, but these technologies are still gaining speed in the clinical functional medicine arena. The EXALT study was the first prospective precision medicine trial to use a personalized functional medicine assay to guide therapeutic decision making. This study employed an image-based single-cell drug screen platform with automated high content microscopy and image analysis (formerly called pharmacoscopy) to test treatment for patients with advanced aggressive hematologic cancers. Fifty-four percent of those treated according to single cell functional precision medicine (scFPM) results experienced 1.3-fold improvements in PFS compared with prior therapies, and 40% of these patients had outstanding responses that lasted 3-fold longer than predicted for their disease (93). This study demonstrates the promise of *in vitro* functional drug screening, though the 3D environment of PDOs and PDXs has the potential to more faithfully recapitulate the disease state and patient response to various therapeutics. Previous studies using a similar single-cell automated imaging assay-guided approach demonstrated feasibility for clinical integration and led to improved treatment outcomes for hematologic malignancies (94, 95).

The Oncogram clinical trial is utilizing the Ongramme chemosensitivity test to provide functional precision medicine to patients; results of functional testing are provided to clinicians within 15 days (NCT03133273) and the assay demonstrated its utility in a pilot study of metastatic CRC (96). A recent trial among relapsed or refractory multiple myeloma patients utilized a 2D CLIA-certified high throughput drug screen (HTS) of 170 compounds coupled with whole exome, mRNA sequencing, and targeted sequencing of circulating tumor DNA. Results for the screen were available within a median of 5 days, and 92% of patients who received treatment based on the functional medicine HTS achieved disease control (97).

### Patient-Derived Organoid Trials

A CLIA certified PDO drug assay by SEngine Precision Medicine recently reported a high concordance of drug sensitivity with known genomic anchors as well as sensitivity to target agents in the absence of known genomic biomarkers (98–100). The observed sensitivity of organoids lacking appropriate biomarkers to targeted therapies underlines the importance of functional testing and its ability to identify therapies for patients with no known biomarkers. A co-clinical trial including rectal cancer organoids demonstrated that PDOs faithfully model the pathophysiology and genetic evolution of their corresponding tumors, and observed highly matched responses with patients to chemoradiation (29). A prospective trial of pancreatic PDOs demonstrated the feasibility of introducing this model to the clinic with 91.1% success in predicting response to first-line regimens among treatment-naïve patients (101). Several ongoing clinical trials are performing drug screens in PDOs to guide treatment decisions in advanced breast cancer (NCT04655573), bladder cancer (NCT05024734), pancreatic cancer (NCT04469556 and NCT05196334), and those with refractory solid tumors (NCT04279509).

### Patient-Derived Xenograft Trials

Many current trials are combining NGS with PDX models to provide personalized functional medicine to cancer patients and study disease progression and therapy resistance. Current clinical trials utilizing NGS and functional drug screening in PDX and PDO models to guide treatment decision making are listed in Table 1. The ongoing Match-R study is combining whole exome sequencing (WES), RNA sequencing, and targeted panel sequencing with PDX models to characterize the molecular mechanisms of resistance to cancer treatments. Thus far, 65% of samples were successfully characterized by targeted NGS (e.g., WES and RNA sequencing), and the success rate for the development of PDX models was 33%. 11/12 PDX models exposed to the same drug as the patient before disease progression replicated the patient response, indicating that selective pressure *in vivo* mimics patient drug resistance (NCT02517892). The Tumorgraft study (NCT02752932) utilized murine PDX models to screen four compounds for each patient. Unfortunately, average time to engraftment was 89.2 days. Of the two eligible living patients who underwent Tumorgraft guided therapy, one patient failed to respond to a therapy that was efficacious in their corresponding xenograft model, and the other patient responded to the compound most potent in their matched xenograft model (102). An Australian study combined NGS with *in vitro* and *in vivo* drug screening to identify therapies for children with high-risk cancer; this comprehensive approach altered the treatment course of 53% of patients, among whom 29% experienced clinical benefit. However, 46% of cultures failed to expand and 43% of PDX’s failed engraftment (103). Evidently, the murine PDX model is still in development and optimization is necessary for continued advancement and implementation of this functional precision medicine approach. Nevertheless, several precision medicine trials coupling NGS with PDX drug screening to guide treatment decision-making are ongoing in biliary tract cancer (NCT02943031), castration resistant prostate cancer (NCT03786848), metastatic androgen dependent prostate cancer (NCT02795650), high grade osteosarcoma (NCT03358628), and pancreatic cancer (NCT04373928).

**TABLE 1:** Implementation of integrative molecular and drug profiling in clinical trials: selected clinical trials utilizing NGS and functional drug screening in PDO and PDX models to guide treatment decision making.

Study title	Cancer type	Molecular profiling	Drug screening description	Primary endpoint (s)	ClinicalTrials.gov identifier

Clinical trials utilizing NGS and drug screening in PDOs to guide treatment decision-making					
Selecting Chemotherapy With High-throughput Drug Screen Assay Using Patient Derived Organoids in Patients With Refractory Solid Tumors (SCORE)	Head and neck squamous cell carcinoma, colorectal, breast, or epithelial ovarian cancer	Gene expression and proteomics analysis	• Invitrocue PDO • 10–15 drug panel screen	Overall radiological response rate and correlation between genotype, tumor biomarkers, and blood biomarkers with clinical outcome	NCT04279509

Functional Precision Oncology for Metastatic Breast Cancer (FORESEE)	Metastatic breast cancer	Genome sequencing	• Drug selection for organoid sensitivity testing is guided by results of genome analysis	Number of cases where clinically actionable outcomes were identified by functional precision oncology approach	NCT04450706

*Ex Vivo* Drug Sensitivity Testing and Mutation Profiling	Relapsed/refractory pediatric cancer patients	Genomic profiling of cancerous and germline tissue	• *Ex vivo* high throughput drug sensitivity testing	Percentage of patients that receive drug sensitivity testing guided treatments	NCT03860376

The PIONEER Initiative: Precision Insights On N-of-1 *Ex Vivo* Effectiveness Research Based on Individual Tumor Ownership (Precision Oncology) (PIONEER)	Any cancer patient	Genomic profiling	• *Ex vivo* drug sensitivity testing in organoids	Return of research information to individual patient and cancer care team over time	NCT03896958

** Clinical trials utilizing NGS and drug screening in PDXs to guide treatment decision-making**					
Personalised Therapy for Metastatic ADPC Determined by Genetic Testing and Avatar Model Generation (AVATAR)	Metastatic pancreatic adenocarcinoma	Exome sequencing	• Treatments chosen from a database of >2,000 drugs	1 year survival	NCT02795650

Personalized Mini-PDX for Metastatic CRPC	Metastatic castration resistant prostate cancer	NGS	• MiniPDX • Single drugs and combinations	Objective response rate evaluated by RECIST	NCT03786848

The Effect of Individualized Precision Therapy Programs in Patients With BTC	Biliary tract cancer	WGS	• Mini-PDX and PDX • Drug choices based on genomics information	Overall survival	NCT02943031

Patient-derived Xenograft (PDX) Modeling to Test Drug Response for High-grade Osteosarcoma	High grade bone and soft tissue sarcoma	Comprehensive genomic and epigenetic analysis	• PDX (and PDO if enough tissue remains)	Ability of PDX drug screen to predict clinical response in matched host	NCT03358628

TumorGraft- Guided Therapy for Improved Outcomes in Head and Neck Squamous Cell Cancer- A Feasibility Study (Xenograft)	(Recurrent metastatic) head and neck squamous cell carcinoma	Exome Sequencing or genomic sequencing	• Up to 4 drugs will be tested on each PDX	To determine the rate of PDX engraftment for HNSCC and R MHNSCC, time to engraftment, percentage of models successfully undergoing drug testing, and participant status upon completion of drug testing	NCT02752932

Personalized Patient Derived Xenograft (pPDX) Modeling to Test Drug Response in Matching Host (REFLECT)	Triple negative breast cancer, colorectal cancer, high grade serous ovarian cancer, and other tumor types	NGS	• Personalized PDX (pPDX) and PDO • Chemo and targeted therapies	Measure of pPDX drug sensitivity as predictor of clinical response in matched host, rate of results reporting and rate of pPDX engraftment	NCT02732860

Personalized Precision Diagnosis and Treatment of Pancreatic Cancer (PPDTPC)	Pancreatic cancer	Not specified (DNA or RNA sequencing)	• Mini PDX/PDX • Chemotherapeutics	Overall Survival	NCT04373928

PRecISion Medicine for Children With Cancer (PRISM) Patient-Derived Xenografts in Personalizing Treatment for Patients With Relapsed/Refractory Mantle Cell Lymphoma	High risk cancers in pediatric and adolescent patients	Targeted whole exon variant analysis, whole genome and transcriptome sequencing, methylation analysis, and proteomics analysis	• *In vitro* high throughput drug sensitivity testing, and PDX testing	Personalized medicine recommendation	NCT03336931

*We searched clinicaltrials.gov for relevant trials using the terms: NGS, sequencing, or drug screening AND organoid, PDO, xenograft, PDX, patient-derived, organ on a chip, OOC, 3D bioprinted, microfluidic, drug screen. We also included trials cited in relevant articles that we judged to be important.*

### Trials With Zebrafish Patient-Derived Xenografts, Organ-on-a-Chip Models, and 3D Bioprinted Models

An observational co-clinical trial is examining the practicality of patient-derived zebrafish xenografts and their ability to predict the most effective chemotherapeutic regimen for cancer patients (NCT03668418). The trial is employing zebrafish avatars to perform a chemosensitivity assessment on each patient, with PDX-guided treatment conclusions available within 1 week of engraftment. There are also two current clinical trials utilizing chick embryos as a host model system for PDX drug testing in those with renal cell carcinoma (NCT04602702) and urogenital malignancies (NCT03551457). The chick chorioallantoic membrane (CAM) PDX model is a lower cost, high throughput alternative to murine models that displays enhanced tumor take rate compared to murine models, conserved tumor heterogeneity, and a strong representation of metastasis; the CAM model is excellently reviewed in Chu et al. (104). One current trial (NCT04996355) is examining the feasibility of drug screening in an OOC model for colorectal cancer and will be comparing tumor response in patients and organoids. Finally, an ongoing study is screening chemotherapy combinations in 3D bioprinted organoids for multiple myeloma patients and validating the predictive capacity of a 3D organoid chemobiogram with retrospective data of donor responses (NCT03890614). Another study is examining the relationship between patient-derived 3D bioprinted model drug sensitivity and patient drug sensitivity amongst colorectal cancer patients (NCT04755907). It remains to be seen whether future trials will incorporate models involving PDXOs.

### Trials With Reprogrammed Cells

Though conditional reprogramming and induced pluripotent stem cell technologies still remain in development, these methods are gaining footage in the clinical trial arena. Currently, one clinical trial is utilizing drug screening in PDX and mini-PDX platforms to guide treatment and will be performing CR on patient cells to follow up on tumor drug susceptibility testing (NCT04373928). An ongoing NCI protocol is combining genetic testing with reprogramming of patient cells to iPSCs in order to generate cancer antigen-specific T cells (NCT03407040). In another trial, iPSCs will be derived from patients who experience cardiotoxicity after thoracic radiation in order to identify mechanisms of radiation-induced cardiotoxicity (NCT04674501). Finally, among neurofibromatosis type 1 population, agents are being screened in patient-derived iPSC lines with different genotypes to identify drugs capable of reversing disease phenotypes (NCT03332030).

### Trials Based on Mathematical Models/Artificial Intelligence

Artificial intelligence is being incorporated into clinical trials at various stages of the treatment process. In the AI-EMERGE study among among CRC patients, plasma was analyzed *via* WGS, bisulfite sequencing, and protein quantification, then a multiomic machine learning algorithm was applied to detect early-stage CRC. This machine learning approach using cell-free DNA demonstrated high specificity and sensitivity for detection of early-stage disease in a CRC cohort (105). Ongoing clinical trials are utilizing AI to locate occult cancer cells for the purpose of improving the future of glioma resection (NCT00330109) and implementing AI to ameliorate radiotherapy in head and neck squamous cell carcinoma patients via the development of adaptive radiotherapy (NCT05081531). Likewise, a current breast cancer study is combining genomics, imaging, and AI to uncover the relationships between disease features, establish predictive models for the sensitivity of HER2 positive breast cancer to targeted therapy, and elucidate the imaging genomic characteristics of the therapeutic targets of each breast cancer subtype (NCT04461990). Another model employed deep learning and the antitumor immune potential of genes to predict response to immunotherapy and was validated in melanoma and metastatic urothelial cancer cohorts (106). Finally, a clinical trial incorporating a transcriptomics-based computational approach to predict patients’ response to various treatments (similar to the SELECT algorithm) will be forthcoming at the National Cancer Institute’s Center for Cancer Research. Despite the major promise that integrated big data holds as a contributor to the precision medicine armamentarium, major structural changes must occur to construct research-ready digital biobanks while data availability remains a major roadblock (107). Emerging techniques such as spatial and single-cell genomics will produce a wealth of powerful data to be processed *via* artificial intelligence in the future.

## Challenges and Looking Ahead

In a study of clinical trial success rates from 2005 to 2015, the overall probability of success for drug development programs across all oncology clinical trial phases (I–III) was 2.1%; for trials using biomarkers, the probability of success was 10.7% (108). Similarly, the UCSD-PREDICT study demonstrated that personalized treatment decisions made by a molecular tumor board with the intent to target the highest number of pathogenic alterations possible significantly improved PFS and overall survival (109). Evidently, precision medicine greatly improves patient outcomes, and expansion of the precision medicine toolbox is necessary to develop the breadth and depth of this effective clinical practice.

The practicality of functional precision medicine poses a major challenge; currently, most resected tissue is rapidly fixed for histology or processed for sequencing, preventing further use for FPM assays. Likewise, hospitals abide by a variety of protocols governing tissue handling after resection; lack of standardization of these processes could ultimately affect the implementation of FPM assays moving forward. Drug concentrations and drug treatment duration in patient-derived models and patients themselves frequently differ (110). Some models, like PDOs, can only inform tumor drug response, excluding potential toxicities to other organs. Patient dosing regimens are also challenging to replicate, though OOC technology is allowing for dosing schedules that mimic the constant infusions some patients experience (57). Furthermore, FPM assays must be optimized such that patients and clinicians can receive therapy-guiding results within a reasonable time frame. The unreliable take rates of PDOs and PDXs must specifically be improved to fit within this window. FPM assays must become standardized, if not automated to provide the high throughput capacity necessary for practical implementation in the clinical realm. The possibility of high throughput 3D printing has been explored (111, 112), and 3D printed organ on a chip technology likewise presents an exciting opportunity for a standardized, automated, and potentially high throughput personalized drug screening platform. Artificial intelligence and machine learning also have a long way to go. Most advancements in AI use one type of data, but deep learning approaches capable of integrating various forms of data will be necessary for holistic precision medicine models. Likewise, medical data is difficult to share between institutions due to confidentiality policies, rendering access to the information necessary for validation and training of new AI technologies difficult (113).

Not only must drug screening join NGS and AI to advance FPM, but combination therapies and mechanisms of resistance must continue to be explored. Coupling FPM assays with molecular/genomic screening can elucidate not only mechanisms of primary and acquired resistance, but more importantly provide insight on how to overcome them. Forthcoming precision medicine trials are focusing on addressing treatment combinations in areas of unmet clinical need. To overcome acquired drug resistance to single-agent therapy, NCI-ComboMATCH, the next phase of the NCI-MATCH trial (which consisted mostly of single agents), is implementing genomically directed targeted agent combinations supported by preclinical PDX and cell line derived xenograft data (114). In the next-generation NCI immunoMATCH (iMATCH) trial, tumors will be classified into subgroups representing biological mechanisms of resistance and prospective biomarker stratification will guide precision medicine. Enrolled patients will undergo immune profile testing to determine their tumor mutation burden and tumor inflammation signature (NCT05136196).

Another FPM technique that can assist precision medicine decision-making is BH3 profiling, which assesses apoptotic priming and is used to predict response to chemotherapy and targeted combinations (115). Apoptosis occurs when pro-apoptotic proteins are activated, homo-oligomerize, and lead to mitochondrial outer membrane permeabilization (MOMP), which commits the cell to apoptosis. The BCL-2 family of proteins regulates commitment to MOMP and cells can be grouped into several classes of readiness, or priming, for apoptosis based on the presence or absence of various BCL-2 family proteins. BH3 profiling can predict clinical response and has successfully been utilized to determine sensitivity to Venetoclax, a BCL-2 inhibitor, and hypomethylating agents in acute myeloid leukemia (116). A high throughput dynamic BH3 profiling (HT-DBP) platform was recently developed that allows for rapid screening of numerous compounds (117). This method is unique because it can identify compounds that prime cells for apoptosis, but may not induce it, potentially enabling the recognition of compounds that would not receive attention by other measures of cell death. Not only is BH3 profiling a practical functional precision medicine technique, but it provides a nuanced metric of drug efficacy. Several clinical trials have incorporated BH3 profiling to assess drug sensitivity for Venetoclax-based combinations (NCT04898894, NCT04512105, NCT03404193, NCT03214562, and NCT03471260).

The ongoing I-PREDICT study assigned personalized combination treatment regimens based on DNA sequencing results, NGS of circulating tumor DNA, PD-L1 status, microsatellite instability status, and tumor mutational burden. I-PREDICT observed that targeting more molecular alterations was correlated with significantly improved disease control rates and longer PFS and overall survival (OS) (compared to those for whom fewer molecular alterations were targeted). Of the 83 patients in this study who received treatment, each molecular profile and most therapeutic regimens were unique, underscoring the need for personalized therapy and the major potential of combination therapies (118). On the note of disease heterogeneity, patient-derived models are frequently initiated using cells from a core needle biopsy which fails to represent intratumoral heterogeneity. Many cancers are multiclonal and mutations that have the potential to define treatment choices could be clonal or subclonal (119, 120). Five different PDO cultures from different regions of the same CRC tumor displayed genetic and transcriptomic heterogeneity as well as a 30-fold difference in drug response, underlining the immensity of intratumoral heterogeneity and its impact on treatment (121). Advancements in computational biology and AI, in addition to data gained from single-cell sequencing and spatial transcriptomics will ultimately enable adapted computer models that can recapitulate intratumoral heterogeneity and the variety of drug responses that occur within a single tumor. Likewise, many patient-derived preclinical models do not allow for study of metastasis and the anti-metastatic effect of therapeutics on an individual’s disease, though improving PDX models and developing human-on-a-chip technology will soon provide models for patient specific metastasis studies.

Once FPM technologies are optimized, accessibility to clinical trials still presents a major hurdle. A study based out of Memorial Sloan Kettering utilizing the MSK-IMPACT comprehensive sequencing assay found that although 37% of participants had clinically actionable alterations, only 11% of the first 5,009 patients tested were enrolled in a clinical trial involving a molecularly targeted agent, reflecting difficulties with geographical accessibility to clinical trials and patient preference (122). In an attempt to overcome limited accessibility to precision oncology treatment, the ongoing PIONEER initiative is focused on providing any cancer patient at any medical facility access to genomic testing and *ex vivo* drug testing (NCT03896958).

## Conclusion

As PDX models continue to evolve, 3D personalized drug screening coupled with NGS is emerging as a multipronged approach to guide treatment decision making. Developing OOC and 3D printed models will provide more ethical alternatives to PDXs for personalized drug screening in the future and the application of ML to data garnered by way of these technologies will uncover a wealth of knowledge in the years to come. Spatial omics is likewise evolving to further elucidate the complexity of the tumor microenvironment. Coupled with AI analysis, spatial omics technology is capable of linking pathology images with spatial gene expression profiles and spatial maps of tumor samples will uncover new potential treatment avenues (123). Single-cell mass analysis has also emerged as a potentially useful functional medicine tool for prediction of drug response and treatment outcome in patient derived neurosphere models (124). Integration of proteomic, epigenomic, transcriptomic, genomic, and functional data is necessary for the identification of new biomarker signatures and the most efficient targeting of the disease at hand (125). As multi-omics technology and drug screening advance, it is crucial that data be shared and updated such that the developing field of AI can continue to improve and integrate the vast quantities of data generated. Furthermore, implementation of comprehensive functional precision medicine testing at various disease stages will allow us to uncover mechanisms of disease progression, metastasis, and treatment resistance and to identify the right treatment for each patient at each specific point of their treatment journey.

## Author Contributions

GN and CC conceived and structured the content. All authors contributed to the writing and editing of the article, and approved the submitted version.

## Author Disclaimer

The content of this publication does not necessarily reflect the views or policies of the Department of Health and Human Services, nor does mention of trade names, commercial products, or organization imply endorsement by the U.S. Government.

## Conflict of Interest

The authors declare that the research was conducted in the absence of any commercial or financial relationships that could be construed as a potential conflict of interest.

## Publisher’s Note

All claims expressed in this article are solely those of the authors and do not necessarily represent those of their affiliated organizations, or those of the publisher, the editors and the reviewers. Any product that may be evaluated in this article, or claim that may be made by its manufacturer, is not guaranteed or endorsed by the publisher.

## Abbreviations

NGS: next generation sequencing
WGS: whole genome sequencing
PDX: patient derived xenograft
pPDX: personalized patient derived xenograft
PDO: patient derived organoid
OOC: organ on a chip
ADPC: adenocarcinoma of the pancreas
CRPC: castration resistant prostate cancer
BTC: biliary tract cancer
HNSCC: head and neck squamous cell carcinoma
RMHNSCC: recurrent metastatic head and neck squamous cell carcinoma.
